# FASTQuick: rapid and comprehensive quality assessment of raw sequence reads

**DOI:** 10.1093/gigascience/giab004

**Published:** 2021-01-29

**Authors:** Fan Zhang, Hyun Min Kang

**Affiliations:** Department of Computational Medicine and Bioinformatics, University of Michigan Medical School, 100 Washington Ave, Ann Arbor, MI 48109, USA; Department of Biostatistics, University of Michigan School of Public Health, 1415 Washington Heights, Ann Arbor, MI 48109, USA

**Keywords:** quality assessment, genetic ancestry, contamination, sequencing data analysis

## Abstract

**Background:**

Rapid and thorough quality assessment of sequenced genomes on an ultra-high-throughput scale is crucial for successful large-scale genomic studies. Comprehensive quality assessment typically requires full genome alignment, which costs a substantial amount of computational resources and turnaround time. Existing tools are either computationally expensive owing to full alignment or lacking essential quality metrics by skipping read alignment.

**Findings:**

We developed a set of rapid and accurate methods to produce comprehensive quality metrics directly from a subset of raw sequence reads (from whole-genome or whole-exome sequencing) without full alignment. Our methods offer orders of magnitude faster turnaround time than existing full alignment–based methods while providing comprehensive and sophisticated quality metrics, including estimates of genetic ancestry and cross-sample contamination.

**Conclusions:**

By rapidly and comprehensively performing the quality assessment, our tool will help investigators detect potential issues in ultra-high-throughput sequence reads in real time within a low computational cost at the early stages of the analyses, ensuring high-quality downstream results and preventing unexpected loss in time, money, and invaluable specimens.

## Findings

### Introduction

Efficient and thorough quality assessment from deeply sequenced genomes on an ultra-high-throughput scale is crucial for successful large-scale sequencing studies. Delay or failure in detecting contamination, sample swaps, quality degradation, or other unexpected problems in the sequencing or library preparation protocol can result in enormous loss of time, money, and invaluable specimens if, e.g., hundreds or thousands of samples are found to be contaminated weeks or months later. Currently, quality control (QC) tools for sequencing data analyses either have to wait hundreds of CPU hours for sequence alignment results to generate comprehensive QC metrics or completely skip the sequence alignment step and ignore alignment information to achieve faster turnaround. A desired strategy that can generate comprehensive QC metrics of sequence data at real-time speed will ensure the generation of high-quality sequence reads and successful outcomes in the downstream analyses.

Existing quality assessment or QC tools mainly fall into 2 categories—pre-alignment and post-alignment methods—on the basis of whether they require full alignment of the genome prior to the quality assessment. Pre-alignment methods, such as FASTQC [1], PIQA [2], and HTQC [3], produce read-level summary statistics that can be obtained from sequence reads, such as base compositions, *k*-mer distributions, base qualities, and GC bias levels. However, these pre-alignment methods do not estimate many key quality metrics required for comprehensive quality assessment. These missing metrics include mapping rate, depth distribution, the fraction of genome covered, sample contamination, or genetic ancestry information. Other post-alignment methods, such as QPLOT [4], Picard [5], GotCloud [6], and verifyBamID [7], provide a subset of these key quality metrics but require full alignment of sequence reads, which typically takes hundreds of CPU hours for deep (e.g., >30×) sequence genome (Table 1).

**Table 1:** Quality assessment metrics provided by different QC tools

Metric	FASTQC	PIQA	HTQC	QPLOT	Picard	verifyBamID2	FASTQuick
Base quality per cycle	✓	✓	✓	✓	✓		✓
GC bias				✓	✓		✓
PCR duplication rate				✓	✓		✓
Insert size distribution				✓	✓		✓
Contamination estimate					✓	✓	✓
Genetic ancestry						✓	✓
% Mapped reads				✓	✓		✓^a^
Depth distribution				✓	✓		✓
Total No. of reads	✓			✓	✓		✓
Read length distribution	✓		✓	✓	✓		✓
Full alignment not required	✓	✓	✓				✓

a Currently only recommended for whole-genome sequencing dataset.

We describe FASTQuick, a rapid and accurate set of algorithms and software tools, to combine the merits of QC tools from both categories. By focusing on a variant-centric subset of a reference genome (reduced reference genome), our methods offer up to 30–100-fold faster turnaround time than existing post-alignment methods for deeply sequenced genome while providing a comprehensive set of quality metrics comparable with QPLOT and verifyBamID (full-alignment–based results from these 2 tools together constitute most of the important QC metrics from the GotCloud-based QC pipeline, which we compare against below) with the help of statistical adjustments to account for the reduced reference genome.

### Computational efficiency

**Figure 1:**
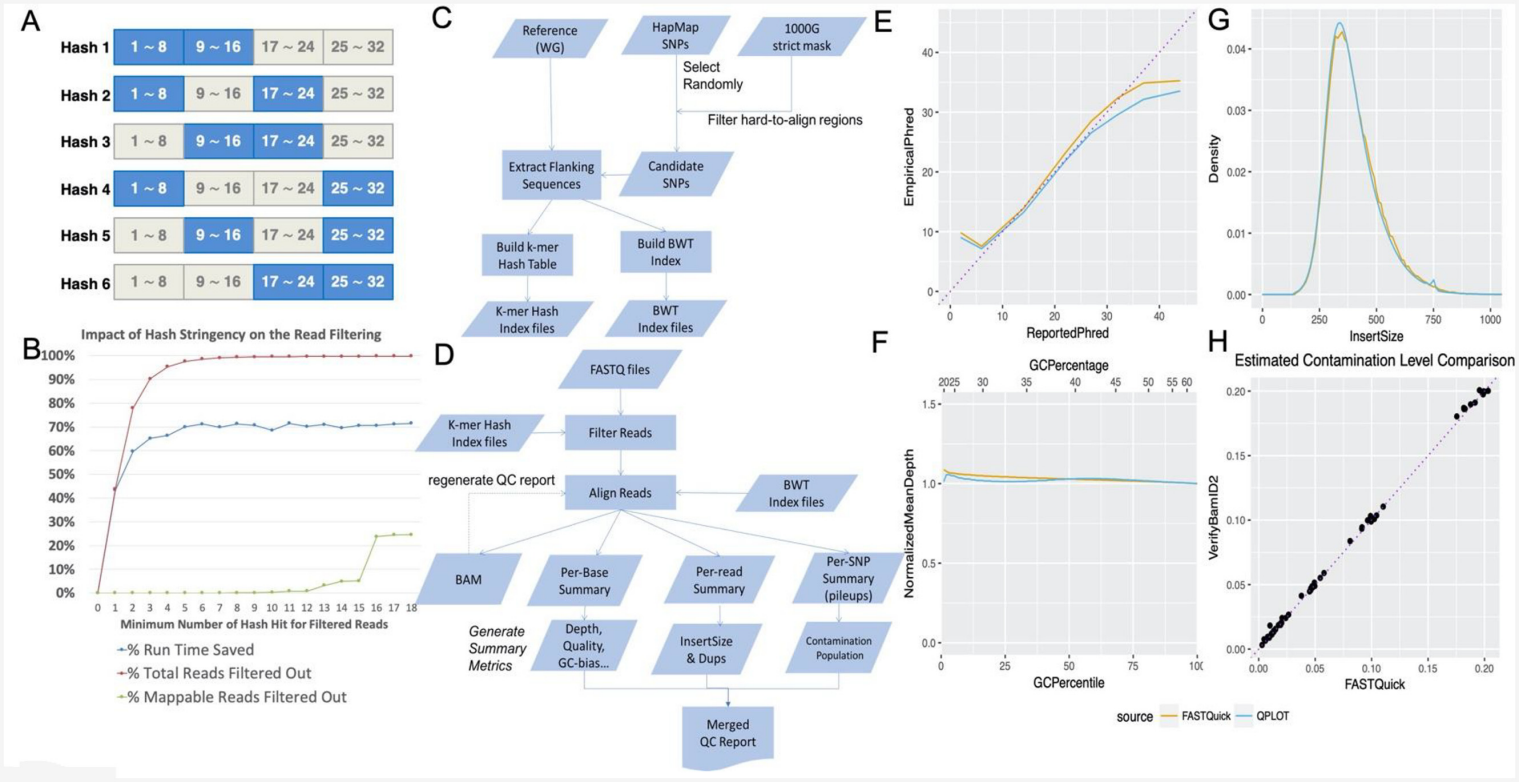
Illustration of FASTQuick. (A) Spaced *k*-mer hash filter design with the tolerance of mismatches for each 32-mer. (B) Effect of minimum spaced *k*-mer hits to be considered for BWA alignment on the overall run time, fraction of total reads filtered, and fraction of falsely filtered alignable reads. *k* = 3 was used in our experiment. (C) Procedure to build FASTQuick indices with a reduced reference genome for spaced *k*-mer hash and the BWA algorithm. (D) Procedure to process sequence reads and produce QC metrics using FASTQuick. (E) Comparison of visualizations of reported base qualities (in Phred scale) and empirical base qualities between QPLOT and FASTQuick for a 38× genome. (F) Comparison of visualization of GC bias (in normalized mean depth) between QPLOT and FASTQuick for a 38× genome. (G) Comparison of estimated insert size distributions between QPLOT and FASTQuick (after Kaplan-Meier adjustment) for a 38× genome. (H) Comparison of estimated contamination rates in *in silico* contaminated 1000 Genomes Project  samples between verifyBamID2 and QPLOT. The purple diagonal dotted line represents y = x.

The primary goal of FASTQuick is to achieve comprehensive QC with much less computational cost than full-alignment–based QC procedures. A large fraction of the computational gains come from the use of the reduced reference genome and filtering of unalignable reads through mismatch-tolerant spaced *k*-mer hashing (Fig. 1A) [8]. Compared to alignment to the full human reference genome, aligning a 3× HG00553 genome on the reduced reference genome reduced the run time by 34.9-fold (94,020 vs 2,697 seconds) using the same algorithm. Using a hash table built from mismatch-tolerant spaced *k*-mers, >90% of unalignable reads can be filtered out with very little loss (Supplementary Table S1) of alignable reads, when ≥3 hits are required (default parameter) for a read to be considered as alignable, saving an additional 65% of computational time (Fig. 1B). Putting them together, the alignment step of FASTQuick (with default parameters) was 100-fold faster (94,020 vs 939 seconds) than the full genome alignment. We observed that >99% of unalignable reads could be filtered out with a more stringent threshold (≥7 hits) at the expense of 0.01% loss of alignable reads. However, the additional computational gain was only 14% (939 vs 811 seconds).

We also evaluated the overall computational efficiency between FASTQuick and the GotCloud-based QC pipeline (typical sequence processing pipeline based on full genome alignment as in 1000 Genomes Project and TOPMed project) on the high-coverage genome (38×) and low-coverage (3×) genomes from the 1000 Genomes Project (Table 2). The results demonstrate that FASTQuick produces a comparable set of QC metrics to GotCloud with a 30–100-fold faster turnaround time.

**Table 2:** Running time comparison

No. of threads	FASTQuick Time (h)		GotCloud QC Time (with BWA) (h)	
	HG00553 (3×)	NA12878 (38×)	HG00553 (3×)	NA12878 (38×)
1	1.03	5.48	30.95	369.56
2	0.53	2.46	21.53	230.85
4	0.33	1.76	15.83	154.91
8	0.24	1.75	12.74	131.85

Running time is evaluated as wall-clock elapsed time on a machine with Intel® Xeon® CPU (X7560 at 2.27 GHz). Reference indexing time is independent of the input sequence dataset and not included. (It takes 3min20s to index human genome under default settings.)

### QC metrics produced by FASTQuick

FASTQuick can automatically generate and visualize the QC metrics listed in Supplementary Table S2. Briefly, FASTQuick generates 3 types of generic QC summary statistics—per-base, per-read, and per-variant summary statistics. Per-base summary statistics inform mapping rate, depth distribution, GC bias, and base quality. Per-read summary statistics allow us to estimate PCR duplication rate and insert size distribution adjusted to account for pair-end alignment bias due to the reduced reference genome. Per-variant summary statistics allow us to estimate DNA contamination rate and genetic ancestry. These summary statistics are combined, jointly analyzed, and visualized into an interpretable and user-friendly quality report shown as in Supplementary Items S1 and S2.

### Accuracy of QC metrics

We compared the distribution of QC metrics generated from FASTQuick with those from GotCloud on multiple sequenced genomes. The QC metrics shared between FASTQuick and GotCloud are listed in Supplementary Table S2. The visualization QC metrics such as base quality recalibration (Fig. 1E), normalized mean depth by GC content (Fig. 1F), and depth distribution are very close between FASTQuick and GotCloud. For example, the 2-sample Kolmogorov-Smirnov (KS) test statistic, which quantifies the maximum differences between 2 empirical cumulative distributions of depth, was D = 0.040. Similarly, the Wasserstein-1D distance, which quantifies the average distance between 2 cumulative distributions of depth, was W = 0.0038. The Wasserstein distance is a widely used metric to evaluate the similarity between 2 distributions in generative adversarial networks [9]. Even though such differences are statistically significant (mainly because of the large number of observations), it is arguably a small amount of difference that is typically observed between different QC tools on the same sequence data. We also evaluated the estimated PCR duplication rate by comparing with QPLOT's result using 10 randomly selected samples from the 1000 Genomes Project, which shows a difference almost within 1.5% (Supplementary Table S3). To further facilitate other potential analyses that require genotype availability, such as relatedness2, we also generated a VCF file that contains GT, PL, and GP fields. The genotype accuracy is ∼99% by comparing with the 1000 Genomes Project phase3 call set (Supplementary Table S4).

One challenge in quality assessment based on the partial alignment of sequence reads to the reduced reference genome is the estimation of insert size distribution. To systematically correct for biased estimation of insert sizes, we statistically integrated the observed insert sizes across all contigs with inverse probability weighting based on the Kaplan-Meier curve [10] (see Methods). Applying our correction produces an estimated insert size distribution much closer to that from the full alignment (Fig. 1G). The KS-test statistic and the Wasserstein-1D distance were D = 0.60 and W = 0.0591, respectively, when using 500-bp contigs only, but they decreased to D = 0.18 and W = 0.0170 when using both 500- and 2,000-bp contigs when comparing the insert size distributions between FASTQuick and GotCloud. When adjusting the insert-size distribution using a Kaplan-Meier estimator, they substantially decreased to D = 0.017 and W = 0.0066, respectively.

To evaluate the estimation accuracy of contamination rate and genetic ancestry, we prepared artificially contaminated 1000 Genomes Project samples *in silico* (see Methods). Then we compared the estimated contamination rate and genetic ancestry from FASTQuick with the estimation from the full-alignment QC pipeline-based result. Our results demonstrate that FASTQuick can estimate contamination rate (Fig. 1H) and genetic ancestry (Supplementary Table S5) as accurately as the standard method VerifyBamID2 relying on the full-alignment result. For example, HG00553(PUR) and NA12878(CEU) are correctly mapped onto their corresponding genetic ancestry group (Supplementary Items S1 and S3).

## Methods

### Overview of FASTQuick

FASTQuick first constructs a reduced reference genome from a set of flanking sequences surrounding known single-nucleotide polymorphisms (SNPs) and builds a Burrows–Wheeler transform (BWT) index [11] and mismatch-tolerant *k*-mer hash table (Fig. 1C). Once the indices are built, FASTQuick rapidly filters out unalignable reads whose first 96 bp have <3 hits (out of 18 potential hits, among which 6 hits per 32-mer) against the spaced *k*-mer hash indices, and aligns filtered sequence reads to the reduced reference genome using the BWT index (Fig. 1D). The small fraction of filtered aligned reads will be stored in binary Sequence Alignment/Map format (BAM) [12]. Next, all the summary statistics that are generated from the aligned reads are collected and jointly analyzed to form various QC metrics that are reported in a user-friendly report in HTML (Supplementary Item S1).

### Construction of reduced reference genome using flanking sequences of SNPs

FASTQuick constructs a reduced reference genome based on well-alignable flanking sequences around known common SNPs to enrich the reads that are informative for both genetic inference (e.g., contamination and ancestry) and other genomic quality metrics that require read alignment. Starting from an arbitrary set of known SNPs, FASTQuick randomly selects a designated number of SNPs from a known common (minor allele frequency >5%) SNP set, such as HapMap3 [13], while excluding SNPs near hard-to-align regions (e.g., 1000 Genomes Project strict mask region). FASTQuick then constructs a reduced reference genome using short flanking sequences of the majority of SNPs (e.g., 90%) and long flanking sequences of the remaining SNPs.

### Filtering unalignable reads with mismatch-tolerant hash

Because the reduced reference genome is a small subset of the whole genome sequence, we expect that only a small fraction of reads will be alignable. However, attempting to align all the reads is still computationally expensive. FASTQuick builds a hash-based index to rapidly filter out the reads that are unlikely to be aligned to the reduced reference genome. To make the hash robust against sequencing errors, FASTQuick builds 6 locally sensitive hash tables of 16-mers for each 32-mer (Fig. 1A) so that 32-mers with ≤2 mismatches can still be guaranteed to match to ≥1 of the hash tables [8].

FASTQuick partitions each sequence read into multiple 32-mers and performs hash lookups for each possible 16-mer. For example, for a 100-bp read, eighteen 16-mers (6 per 32-mer) across three 32-mers will be matched to the hash table. For reads longer than 96-bp reads, only the first 96-bp reads are used. FASTQuick's decision whether to filter out a read is based on whether the number of matching 16-mers is less than a certain threshold *k*. For example, if *k* is 3, reads with <7 mismatches are guaranteed to pass the filter, and many other reads with more mismatches will pass the filter. If *k* is 10, reads with <3 mismatches are guaranteed to pass the filter. We chose *k* = 3 on the basis of empirical observations (see Findings). The remaining reads will then be aligned by the optimized BWA-like algorithms to the reduced reference genome.

### Generating base-level, read-level, and variant-level QC metrics

Using the reads aligned to the reduced reference genome, FASTQuick generates a full list of base-level, read-level, and variant-level QC metrics (Supplementary Table S2). Base-level metrics, such as base quality, and sequencing cycle, are recorded directly without using the alignment information. Because the reads spanning the end of flanking sequences may be poorly aligned, FASTQuick produces metrics only on the fully alignable portion of flanking sequences. Let the length of the flanking sequence be *w* and the read length be *r*; then only 2 × (*w*−*r*) +1 bases spanning the variant site will be considered when calculating base-level summary statistics. Read-level QC metrics, such as the fraction of mapped reads and insert size distribution, are estimated and reported on the basis of the read alignment result. Variant-level metrics are collected after the alignment result becomes available and are reported as pile-up bases, estimation of contamination level, and genetic ancestry.

### Bias-corrected estimation of insert size distribution

The insert size distribution is typically estimated from distances between the aligned pairs of reads from the fully aligned reads. When using a reduced reference, a large proportion of paired reads may not be fully mapped, and the read pairs that have shorter insert sizes are more likely to be mapped on both ends. As a result, estimating insert size distribution based only on the reads where both ends are mapped will result in biased estimates of insert sizes, as empirically demonstrated using the 38× genome (Supplementary Figs S1 and S2).

We first attempted to resolve this challenge by extending 10% of the variant-centric contigs to be sufficiently long (2,000 bp) and by estimating insert size only from the reads mapped to longer contigs. This way, we prevent the reduced reference genome from becoming too large to achieve computational efficiency and reduce insert size estimation at the same time. But due to the limited number of long-flanking variants, bias and fluctuations still exist in the estimated insert size distribution (Supplementary Fig. S3).

To infer insert size distribution more accurately, FASTQuick further corrects for the bias nonparametrically using the Kaplan-Meier estimator. Owing to the limited length of flanking sequences in the reduced reference, the observed distribution of insert sizes obtained from the reads that have both ends mapped will be biased towards smaller values. To recover the full distribution of insert sizes adjusting for the “censored” reads (i.e., reads with only 1 of the paired ends aligned) enriched for large insert sizes, we adopted the Kaplan-Meier estimator as an inverse-probability-of-censoring weighted average [10] as described below.

Specifically, we define a tuple (${t_o}$, ${t_l}$, ${t_r}$) (Supplementary Fig. S1) for each mapped DNA segment (or read pair), where ${t_o}$ is the observed insert size, ${t_l}\ $is the maximal insert size of read 1, and ${t_r}$ is the maximal insert size of read 2. The maximal insert size is defined as the distance between the leftmost/rightmost base of read 1/read 2 and the rightmost/leftmost base of the flanking region sequence, respectively. This tuple is fully specified only when a read pair is properly aligned; otherwise, for a single-end mapped read pair (including partially mapped pairs) only 1 of the 2 maximal insert sizes (${t_l}\ $ or ${t_r}$) is available and the unobserved value is set to missing; the rest of the read pairs, such as those that are mapped to different contigs, with low mapping quality, or in abnormal orientation, are discarded in the estimation of insert size distribution. Empirically, given *N* properly aligned read pairs (i.e., tuples without missing values), we can estimate insert size by counting the frequency of different observed insert sizes, ${t_o}$, and the cumulative distribution of insert size hence becomes:

\begin{equation*} F\ \left( t \right) = \frac{1}{N}\ \mathop \sum \nolimits_{i\ = \ 1}^N I\left[ {{t_{o,i}} \le t} \right]. \end{equation*}

However, as mentioned above, this direct estimation will be severely biased because reads mapped only on a single end are more likely to have larger insert sizes. To correct for this bias, we use an approach analogous to the estimation of survival function as $S\ ( t ) = \ 1 - F( t )$. We can view the leftmost/rightmost base on each flanking region as the start time point, the exact insert size ${t_o}$ as the time when it fails to observe the data point, and the maximal insert size, ${t_l}$ and ${t_r}$, as the time when the data point is censored. Let the ordered observed time points ${t_o}$ and censored time points ${t_l}$ (or ${t_r}$) be $\tau $. Denote ${o_t}$ as the number of observed failure cases, i.e., the number of read pairs that have observed insert size ≤*t*, and also denote ${c_t}$ as the number of censored cases at time *t*, i.e., the number of single-end mapped read pairs have maximal insert size ≤*t*, and then let $I[ {{\tau _j} \ge t} ]$ be the indicator function if the *j*th time point is larger than a certain time *t* (*j*th insert size ≥*t*). Then the risk set is:

\begin{equation*} Y\ \left( t \right) = \mathop \sum \nolimits_{j = 1}^J \left( {{o_j} + {c_j}} \right)I\left[ {{\tau _j} \ge t} \right].\
\end{equation*}

Then the Kaplan-Meier estimator $\widehat {{S_{\mathrm{km}}}}$ of $S( t )$:

\begin{equation*} \widehat {{S_{\mathrm{km}}}}\ \left( t \right) = \mathop \prod \nolimits_{\left\{ {j|{\tau _j} \le t} \right\}} \left[ {1 - \frac{{{n_j}}}{{Y\left( {{\tau _j}} \right)}}} \right].\
\end{equation*}

Satten et al. [10] proposed a simplified algorithm to iteratively estimate survival functions for failure times and survival functions for censoring times, by which we conveniently estimate $F( t )$.

### Estimation of contamination rates and genetic ancestry

We also implemented likelihood-based methods to estimate the genetic ancestry and contamination rate in FASTQuick using sequencing data that are mapped onto a random subset of SNPs from the 1000 Genomes Project [14]. The details of these methods have been fully described in VerifyBamID2 [15]. In FASTQuick, to seamlessly integrate these methods into our ultra-fast QC procedure, we designed compatible variant-centric data structures and input/output interfaces that can directly deliver sequence information and estimated statistics from FASTQuick to modules that estimate contamination and genetic ancestry.

### Support for target sequencing dataset

FASTQuick also has provided options to incorporate target regions. We can conveniently use the exome region list for exome sequencing (and it potentially can be extended to an abundantly expressed gene list for RNA sequencing with additional effort to adjust for data type–specific artifacts and biases) as input information to only select markers within the list. We prepared the result generated by FASTQuick for exome-sequencing data of HG00553 from the 1000 Genomes Project as a demonstration (Supplementary Item S2).

## Discussion

We describe FASTQuick, which addresses computational challenges in QC of ultra-high-throughput sequence data, by focusing on sequence reads mappable to an informative subset of the reference genome. Our results demonstrate that FASTQuick achieves on average a 30–100-fold faster turnaround time than methods based on full sequence alignment while producing comprehensive and accurate QC metrics. Compared with previous quality assessment methods that do not align sequence reads at all, FASTQuick provides more comprehensive QC metrics such as depth distribution, insert size distribution, contamination, and genetic ancestry.

FASTQuick leverages several methods, such as spaced *k*-mer hash table and Kaplan-Meier estimator, to enable rapid and accurate estimation of QC metrics. Interestingly, the computational time is much faster than the time required to convert and compress Illumina's BCL formatted files into FASTQ files. Therefore, FASTQuick can work as a UNIX pipe during the conversion procedures to increase efficiency in the sequencing pipeline.

There are potential drawbacks of only using the reduced (subset of) reference genome, but FASTQuick applies heuristics to avoid such drawbacks. For example, reads that originate from multiple homologous regions on the genome may be misaligned to the same contig on the reduced genome, which may affect variant-level quality metrics. FASTQuick addresses this issue by strictly selecting regions that are unique and easy to align (callable regions), and we demonstrated the effectiveness by showing that contamination and genetic ancestry estimates are almost identical to the estimation from the full genome alignment result. Another issue could be the excessive single-end alignment. For example, it will skew the estimation of the insert size distribution toward a smaller value. We applied a Kaplan-Meier estimator to correct the estimation as described above. There are still limitations associated with the reduced reference genome. For example, a precise estimation of percentage of mapped reads is challenging, especially for targeted sequencing reads, owing to the lack of repetitive sequences. Analysis involving structural variation or comprehensive screening of genome-wide association study variants may not be feasible under FASTQuick’s settings.

Currently, FASTQuick is only suitable for short sequence reads. To enable an analysis of long sequence reads, additional alignment algorithms such as Minimap2 [16] could be incorporated. Extending FASTQuick to other types of sequence data, such as RNA sequencing, chromatin immunoprecipitation followed by sequencing, and assay for transposase-accessible chromatin using sequencing should also be possible if the technology-specific characteristics are properly considered and accounted for. In addition, FASTQuick can serve as a general down-sampling step prior to analysis like sample-swap detection or kinship estimation with the help of alignment results on common variants. More broadly, although we demonstrated FASTQuick’s capability by using human genome analysis as an example, the whole pipeline is easily adaptable to other organisms for which corresponding genomic databases are available.

Unlike hardware-accelerated solutions that achieve fast speed by introducing specialized hardware, such as DRAGEN [17] and Parabricks [18], FASTQuick gains its speed from optimized algorithms that are specially designed for the reduced genome setting. Compared to omni-purpose proprietary tools like DRAGEN and Parabricks, FASTQuick is an open-source tool that does not require specific hardware such as GPU or field-programmable gate array devices and is specifically designed for quality assessment, which can be critical to attain rapid turnaround time in sequence analysis workflows and add great value to the existing sequence analysis ecosystem.

## Availability and Requirements

Project name: FASTQuick

Project home page: https://github.com/Griffan/FASTQuick

Operating system(s): Linux, MacOS

Programming language: C++, Shell, R

Other requirements: CMAKE, libhts, ggplot2, knitr

License: MIT

RRID:SCR_019269

## Data Availability

Datasets are publicly available at the Trans-Omics Precision Medicine (TOPMed) project [19] and the 1000 Genomes Project [14, 20]. Snapshots of the code, reports, and other supporting data are available from the *GigaScience* GigaDB repository [21].

## Additional Files

**Figure S1**. Definition of insert size tuple. The blue portion represents a reference genome backbone. The orange portion represents the extracted flanking region. The yellow portion represents a variant. The gray bars represent a pair of reads aligning to this flanking region.

**Figure S2**. Marginal distribution of maximum insert size and observed insert size in the reduced genome under 250 bp (short) and 1,000 bp (long) flanking length configuration. (Top) Marginal distribution of maximum insert size. (Right) Marginal distribution of observed insert size (green), along with true insert size distribution (blue) and adjusted insert size distribution (red). (Bottom) Scatter plot of read pairs with maximum insert size and observed insert size being coordinates. Blue dots represent read pairs mapped to the long flanking region; purple dots represent read pairs mapped to the short flanking region. The band between the line “y = x” and line “y = x + 150” shows read pairs that are partially mapped. The line “y = 2x − 500” and line “y = 2x − 2,000” are the effective boundaries where read pairs have both ObservedInsertSize and MaxInsertSize for 250- and 1,000-bp flanking region, respectively.

**Figure S3**. Biased insert size distribution in reduced genome under 250 bp (short) or 1000 bp (long) flanking length configuration. Each color represents 1 scenario of insert size estimation without correction. “Observed.LongRegion” (green) is when insert size distribution is estimated only using reads mapped to the long flanking region; “Observed.ShortRegion” (blue) is when only using reads mapped to the short flanking region; “True” (purple) is insert size distribution estimated under full genome alignment; “Adjusted” (red) is insert size distribution estimated by FASTQuick.

**Item S1**. Detailed quality assessment final report of HG00553 whole-genome dataset

**Item S2**. Detailed quality assessment final report of HG00553 exome dataset

**Item S3**. Detailed quality assessment final report of NA12878 whole-genome dataset

**Table S1**. Impact of mismatch threshold on *k*-mer-hash–based read filtering

**Table S2**. Summary statistics and visualization items produced by FASTQuick

**Table S3**. Estimation of PCR duplication rate on randomly selected 1000 Genomes Project samples

**Table S4**. Genotype comparison summary of sample HG00553 between FASTQuick and 1000 Genomes Project

**Table S5**. Comparison of genetic ancestry estimation between FASTQuick and VerifyBamID2

## Abbreviations

bp: base pairs; BWA: Burrows-Wheeler Aligner; BWT: Burrows–Wheeler transform; CPU: central processing unit; GC: guanine-cytosine; GPU: graphics processing unit; KS: Kolmogorov-Smirnov; QC: quality control; SNP: single-nucleotide polymorphism.

## Competing Interests

The authors declare that they have no competing interests.

## Funding

This work was supported by HL137182 (to H.M.K. and F.Z.), HL117626, and MH105653 (to H.M.K.).

## Authors’ Contributions

F.Z. contributed to the coding material and experiments. Both authors wrote the manuscript and approved the final version of the manuscript.

## Supplementary Material

giab004_GIGA-D-20-00165_Original_Submission

giab004_GIGA-D-20-00165_Revision_1

giab004_Response_to_Reviewer_Comments_Original_Submission

giab004_Reviewer_1_Report_Original_Submission Simon Andrews -- 6/30/2020 Reviewed

giab004_Reviewer_2_Report_Original_Submission Tobias Rausch -- 8/25/2020 Reviewed

giab004_Reviewer_2_Report_Revision_1 Tobias Rausch -- 12/10/2020 Reviewed

giab004_Supplemental_Files
