# Supplementary material for: FASTQuick: rapid and comprehensive quality assessment of raw sequence reads
Source: Gigascience. 2021 Jan 29;10(2):giab004. doi: 10.1093/gigascience/giab004 (PMC7844880; doi:10.1093/gigascience/giab004)
Supplement: giab004_Supplemental_Files [file giab004_supplemental_files.zip › Item_S2_FASTQuick_HG00553_exome.FinalReport.html]

FASTQuick Summary Report


# FASTQuick Summary Report

- FASTQ File List
- Data Production by FASTQ file
- Depth Distribution
- Summary Statistics
- Summary Plot
- Genetic Ancestry Plot

## FASTQ File List

FASTQ List Table

| FileIndex | PairEnd1 | PairEnd2 |
| --- | --- | --- |
| 1 | SRR070481\_1.fastq.gz | SRR070481\_2.fastq.gz |
| 2 | SRR070780\_1.fastq.gz | SRR070780\_2.fastq.gz |

## Data Production by FASTQ file

Data Production Table

| FileIndex | NumOfBases | NumOfReads | NumOfUmappedReads | NumOfLowMAPQReads | NumOfQCPassReads | ReadLength |
| --- | --- | --- | --- | --- | --- | --- |
| 1 | 5496822400 | 54968224 | 1995986 | 640791 | 2336167 | 100 |
| 2 | 5462980800 | 54629808 | 1986680 | 676236 | 2329458 | 100 |
| Total | 10959803200 | 109598032 | 3982666 | 1317027 | 4665625 | 100 |

## Depth Distribution

```
## Warning: Removed 1 rows containing missing values (geom_path).
```

## Summary Statistics

Summary Statistics

| Statistics | Value |
| --- | --- |
| Estimated Read Mapping Rate | 0.458461 |
| Estimated Read PCR Duplication Rate | 0.0657153[212526/3.23404e+06] |
| Whole Genome Coverage | 3.49322[10959803200/3137454505] |
| Expected Read Depth | 234.754[10959803200/46686362] |
| Estimated Read Depth | 107.626[253907945/2359179] |
| Reduced Genome Size | 2359179 |
| Depth 1 or above position fraction | 0.994344 |
| Depth 2 or above position fraction | 0.989347 |
| Depth 5 or above position fraction | 0.974713 |
| Depth 10 or above position fraction | 0.949789 |
| Q20 Base Fraction | 0.971189 |
| Q30 Base Fraction | 0.88142 |
| Estimated AvgDepth for Q20 bases | 105.119 |
| Estimated AvgDepth for Q30 bases | 95.4029 |
| Median Insert Size(>=500bp) | 528 |
| Median Insert Size(>=300bp) | 340 |
| Contamination Level | 8.33904e-05 |

## Summary Plot

```
## Warning: Removed 1 rows containing missing values (geom_path).
```

## Genetic Ancestry Plot
