# Supplementary material for: FASTQuick: rapid and comprehensive quality assessment of raw sequence reads
Source: Gigascience. 2021 Jan 29;10(2):giab004. doi: 10.1093/gigascience/giab004 (PMC7844880; doi:10.1093/gigascience/giab004)
Supplement: giab004_Supplemental_Files [file giab004_supplemental_files.zip › Item_S3_FASTQuick_NA12878_low_coverage.FinalReport.html]

FASTQuick Summary Report


# FASTQuick Summary Report

- FASTQ File List
- Data Production by FASTQ file
- Depth Distribution
- Summary Statistics
- Summary Plot
- Genetic Ancestry Plot

## FASTQ File List

FASTQ List Table

| FileIndex | PairEnd1 | PairEnd2 |
| --- | --- | --- |
| 1 | SRR622461.filt.fastq.gz | SRR622461.filt.fastq.gz |
| 2 | SRR622461\_1.filt.fastq.gz | SRR622461\_2.filt.fastq.gz |

## Data Production by FASTQ file

Data Production Table

| FileIndex | NumOfBases | NumOfReads | NumOfUmappedReads | NumOfLowMAPQReads | NumOfQCPassReads | ReadLength |
| --- | --- | --- | --- | --- | --- | --- |
| 1 | 104199882 | 1031682 | 39053 | 1980 | 1898 | 101 |
| 2 | 18176507824 | 179965424 | 7960537 | 150174 | 363232 | 101 |
| Total | 18280707706 | 180997106 | 7999590 | 152154 | 365130 | 101 |

## Depth Distribution

## Summary Statistics

Summary Statistics

| Statistics | Value |
| --- | --- |
| Estimated Read Mapping Rate | 0.880477 |
| Estimated Read PCR Duplication Rate | 0.0810656[12738/157132] |
| Whole Genome Coverage | 5.8266[18280707706/3137454505] |
| Expected Read Depth | 6.30296[18280707706/2900338372] |
| Estimated Read Depth | 5.54961[25584180/4610085] |
| Reduced Genome Size | 4610085 |
| Depth 1 or above position fraction | 0.985836 |
| Depth 2 or above position fraction | 0.952765 |
| Depth 5 or above position fraction | 0.618347 |
| Depth 10 or above position fraction | 0.08319 |
| Q20 Base Fraction | 0.955341 |
| Q30 Base Fraction | 0.914329 |
| Estimated AvgDepth for Q20 bases | 5.37794 |
| Estimated AvgDepth for Q30 bases | 5.14707 |
| Median Insert Size(>=500bp) | 541 |
| Median Insert Size(>=300bp) | 386 |
| Contamination Level | 0.00173443 |

## Summary Plot

```
## Warning: Removed 1 rows containing missing values (geom_path).
```

## Genetic Ancestry Plot
