# Supplementary material for: FASTQuick: rapid and comprehensive quality assessment of raw sequence reads
Source: Gigascience. 2021 Jan 29;10(2):giab004. doi: 10.1093/gigascience/giab004 (PMC7844880; doi:10.1093/gigascience/giab004)
Supplement: giab004_Response_to_Reviewer_Comments_Original_Submission [file giab004_response_to_reviewer_comments_original_submission.pdf]

Author's Response To Reviewer Comments

Close

Dear Dr. Edmunds,

Thank you for giving me the opportunity to submit a revised version of my manuscript: “FASTQuick: Rapid and comprehensive quality assessment of raw sequence reads" to GigaScience. I appreciate your valuable feedback together with reviewers’ insightful comments on my manuscript. In this updated version, I have made changes to reflect the majority of the suggestions provided by reviewers. Below is a point by point response to reviewer’s comments and suggestions.

Comments from Reviewer #1:

Thanks for your valuable comments, please find my response below where modifications are quoted and tagged with line number.

1. When the authors describe being able to measure contamination it's not completly clear what types of contamination they are hoping to identify. My initial impression would be that it would be contamination from other species or with primers / adapters etc, but from the description it seems that the reduced method would really only robustly identify contamination from other individuals, or potentially other very closely related species (close enough to align to human). Is this correct?

Yes, the type of contamination we are trying to address is cross-sample contamination within the same species. We now clarified this in abstract Findings(L36):

“Our methods offer orders of magnitude faster turnaround time than existing full alignment-based methods while providing comprehensive and sophisticated quality metrics, including estimates of genetic ancestry and cross-sample contamination.”

2. The authors mention using the tool for RNA-Seq (L247) by suggesting the use of abundantly expressed genes. There are potential problems with this approach where some artefacts and biases affecting this type
of data would be difficult to correct with a reduced genome approach (eg rRNA contamination). They do later mention that further work would be required for other techniques (L307) but not in the initial section.

Thank you for pointing this out. The necessity to further configurate the pipeline to address the potential problems that could arise from RNA-Seq has been explicitly described in the initial section(L312):

“FASTQuick also has provided options to incorporate target regions. We can conveniently use the exome region list for Exome-seq (and potentially can be extended to an abundantly expressed gene list for RNA-seq with additional effort to adjust for data type-specific artifacts and biases) as input information to only select markers within the list.”

3. One point which isn't particularly clear is why it is useful to avoid the whole dataset alignment for the initial QC. My presumption would be that all of these datasets would eventually be aligned against a reference genome anyway, so what is the motivation for delaying this alignment step. Is this to be able to reject libraries without having to align them, or for cases where an alignment free assembly is being done? Setting out the rationale a little more clearly in the introduction would help clarify this.

The motivation to screen out defective samples as early as possible consists of two parts: 1) to avoid time consuming downstream analyses (sequence alignment or de novo assembly could consume large amount of CPU hours and is the main bottleneck in WGS pipelines); 2) in large scale sequencing studies, where hundreds of thousands samples are processed in bulk, a timely feedback on QC result could help us detect potential systematic problems in experiment settings in time so that no time and sample will

be wasted.

We have added this part into the introduction section to describe the motivation more clearly(L57):

“Delay or failure in detecting contamination, sample swaps, quality degradation, or other unexpected problems in the sequencing or library preparation protocol can result in enormous loss of time, money, and invaluable specimens if, for example, hundreds or thousands of samples are found to be contaminated weeks or months later. Currently, quality control (QC) tools for sequencing data analyses either have to wait hundreds of CPU hours for sequence alignment results to generate comprehensive QC metrics or completely skip sequence alignment step and ignore alignment information to achieve faster turnaround speed.

4. I had some practical difficulty getting the software to run on our cluster (CentOS 7 based). Firstly, in the quickstart instructions there wasn't any mention of the pre-requisites for the software (they are in the paper and are mentioned slightly later in the full instructions), putting these up front would seem to make sense. On our system we didn't have HTSLIB in the main system library directories so the build failed. There were instructions to add these paths at build time, and this helped, but the build then failed because (I think) the cmake files do not include the -std=c++11 option
required to enable the 2011 C++ standard on GCC 7.3.0 (the default compiler on CentOS 7). I'll submit a bug report to the github repository with a full log, but it would help to more clearly state the dependencies up front and to make the compile work out of the box with something as standard as gcc 7 (or clearly state if this is not possible).

Thanks for the suggestions, I have rearranged the readme page to reflect these changes.
I have moved pre-requisites section up front before the quick start section.
The “-std=c++11” problem is essentially related to an old version of CMake which couldn’t recognize a later introduced command “set(CMAKE_CXX_STANDARD 11)”. I have put both of these two ways of specifying c++11 standard to the cmake files. The installation procedure has been tested on gcc 5, gcc 7, gcc 10 and clang 11.

Comments from Reviewer #2:

Thanks for your valuable comments, please find my response below where modifications are quoted and tagged with line number.

* The abstract would gain in appeal by clearly stating the current major use case of FASTQuick, which is in my opinion human short-read WGS/WES data. In theory, the method could be adapted to non-human species, long reads or other protocols (ATAC-Seq, RNA-Seq, targeted data, ...) but it remains very unclear how much engineering is required for that.

Thanks for pointing out this, we have adjusted the abstract Findings section (Line 34 - 36):

“We developed a set of rapid and accurate methods to produce comprehensive quality metrics directly from a subset of raw sequence reads (from whole-genome or whole-exome sequencing) without full alignment. Our methods offer orders of magnitude faster turnaround time than existing full alignment-based methods while providing comprehensive and sophisticated quality metrics, including estimates of genetic ancestry and cross-sample contamination.”

* The ancestry inference lacks some method details. Is this based on a random subset of 1000 Genomes SNPs or is it using a set of ancestry informative markers (AIMs)? I expect established AIMs to be more robust compared to an arbitrary subset of SNPs for samples outside of 1000 Genomes. An evaluation on clinical samples of known ancestry
would be more informative than the current comparison to 1000 Genomes in Table S3.

The ancestry inference methods are described in our other paper that specifically discussed this method:” Ancestry-agnostic estimation of DNA sample contamination from sequence reads”.

The marker set that we used for demonstration are randomly selected based on a subset of 1000 Genomes SNPs. We agree that a well-established AIMs should be more robust in terms for the ancestry inference, however, AIMs could limit the flexibility of the FASTQuick pipeline on use cases where target regions are specified.
We provided ItemS3 to demonstrate the ancestry inference of NA12878(aka HG001) which is well known to be CEU ancestry. In ItemS3 we correctly mapped NA12878 into CEU cluster.

Hence, we modified Accuracy of QC Metrics section to reflect the change (Line 164 -168):

"Our results demonstrate that FASTQuick can estimate contamination rate (Figure 1H) and genetic ancestry (Table S5) as accurate as the standard method VerifyBamID2 relying on the full-alignment result. For example, HG00553(PUR) and NA12878(CEU) are correctly mapped onto their corresponding genetic ancestry group (Item S1, Item S3)."

and also, in Line 302 – 305:

"We also implemented the likelihood-based methods to estimate genetic ancestry and contamination rate in FASTQuick using sequencing data that are mapped onto a random subset of SNPs from the 1000 Genomes Project. The details of these methods have been fully described in VerifyBamID2[14]."

* PCR duplicates are a major problem for poor quality or low-input samples and the duplicate rate often determines whether a sample can be sequenced deeper or not. This is an essential QC metric and a comparison of this experimental feature of FASTQuick to Picard or QPLOT would strengthen the manuscript.

We have improved the procedure of estimation of PCR duplication rate in our pipeline.

The result evaluation has been added to Table S3 (Line 142-146):

"We also evaluated the estimated PCR duplication rate by comparing with QPLOT's result using 10 randomly selected samples from the 1000 Genomes Project, which shows a difference almost within 1.5% (Table S3)."

* The introduction briefly touches upon sample swaps. I frankly expected to see a genotyped VCF file for the selected SNPs in the output of FASTQuick that users can then further analyse with tools such as vcftools relatedness2 to identify sample swaps. This would be a useful feature to add, especially if the random SNP set is large enough to distinguish unrelated, parent-child, siblings and identity relationships (i.e., matched tumor-normal genomes in cancer studies).

Thank for this suggestion, we agree that a genotyped VCF file could potentially facilitate more analyses. We implemented this feature (VCF file reporting GT, PL and GP) and added in the evaluation of genotyping accuracy in Table S4.(Line 146 - 149)

"To further facilitate other potential analyses that require genotype availability, such as relatedness2, we also generated a VCF file that contains GT, PL, and GP fields. The genotype accuracy is around 99% by comparing with the 1000 Genome Project phase3 call set (Table S4)."

* Please clarify in the manuscript what type of contamination FASTQuick can identify. I believe it is cross-sample contamination but not tumor-in-normal contamination or microbial sample contamination?

Yes, the type of contamination we aim to address is cross-sample contamination within the same species. Modifications have been made to the abstract section to reflect this change (Line 36 - 38)

"Our methods offer orders of magnitude faster turnaround time than existing full alignment-based methods while providing comprehensive and sophisticated quality metrics, including estimates of genetic ancestry and cross-sample contamination."

* Minor issues I noticed while running the software are below. For new users it might help to provide the reference files (dbSNP, hs37d5) already in the right format as a resource bundle for download.
(a) Options are called --fastq_1 and --fastq_2 but readme states
fastq1 and fastq2

(b) dbSNP reference file needs to be bgzip compressed + tabix-indexed
but downloaded file is gzip compressed
(c) hs37d5.fa needs to be bwa-indexed
(d) The output prefix is not used for FinalReport.html
(e) It wasn't clear to me how to run FASTQuick in a UNIX pipe as suggested in the discussion.

Thank you very much for these suggestions. We have fixed all of them accordingly. As for UNIX pipe usage, in an ideal setting, we can run FASTQuick directly as a downstream step of BCL2FASTQ tool, but this requires more configuration in both sides to work.

* On some test samples, the reported QC results were mostly in-line with our standard QC tools (FastQC, Alfred, VerifyBamID, vcftools relatedness2). Remaining issues:
(a) There is an apparent mismatch between the estimated read mapping rate and the reported number of unmapped reads (NumOfUnmappedReads) in the html produced by the software. For instance on one sample, I got 5% unmapped reads but ~0.99 mapping rate (the latter is the correct estimate).
(b) The accessible genome size was totally off sometimes for WGS human samples: "Total Accessible Genome Size 4607026"
(c) It is unclear to me how to interpret "Estimated Percentage of Accessible Genome Covered -4.00405e+14%"
(d) I often got values > 1 for Depth1, Depth2 ... fractions. Please clarify.

a) Because of the reduced reference setting, the estimated read mapping rate is extrapolated by using mapped bases on flanking regions, however the NumOfUnmappedReads reports the actual number of unmapped reads in this setting.
b) Here we mean to report the reduced genome size which is the total size of the flanking regions.
c) and d) There was a bug that related to 0-Depth positions which is now fixed.

* Minor typos
(a) We chose k=3 based on our experiment based on empirical observations
(b) y=2-2000 should be y=2x-2000 (S1 caption)

Thanks, the typos have been fixed.

* The order of S1 and S3 should be changed because S3 introduces the concept of MaxInsertSize that is used in S1.

Yes, we agree that S3 should be introduced early and adjusted accordingly.

* The Figure font size in Figure 1 is too small to be readable.

We have adjusted the Figure 1 layout to improve the font size.

Close
