# Supplementary material for: FASTQuick: rapid and comprehensive quality assessment of raw sequence reads
Source: Gigascience. 2021 Jan 29;10(2):giab004. doi: 10.1093/gigascience/giab004 (PMC7844880; doi:10.1093/gigascience/giab004)
Supplement: giab004_Reviewer_1_Report_Original_Submission — Simon Andrews -- 6/30/2020 Reviewed [file giab004_reviewer_1_report_original_submission.pdf]

Reviewer Report

Title: FASTQuick: Rapid and comprehensive quality assessment of raw sequence reads

Version: Original Submission **Date:** 6/30/2020

Reviewer name: Simon Andrews

Reviewer Comments to Author:

Zhang et al describe FASTQuick, an optimised QC program for (primarily) human whole genome sequencing. Their software employs the use of a reduced genome to dramatically speed up the process of estimating relevant genome statistics by avoiding the necessity for an entire genome alignment at the QC stage.

The paper is well written in clear language. The software is directly available on github under a suitable license, and is well documented.

I have relatively few comments to make regarding this paper as the rationale for the tool is very well described, the description of the processes used to achieve the documented speed increases is very good and whilst I had a number of questions as I read the paper these were all addressed and either accounted for or acknowledged as limitations of their methods. The comparison with other tools is convincing and the methods appear to be robust.

I have only a few minor and practical observations to make;

1. When the authors describe being able to measure contamination it's not completly clear what types of contamination they are hoping to identify. My initial impression would be that it would be contamination from other species or with primers / adapters etc, but from the description it seems that the reduced method would really only robustly identify contamination from other individuals, or potentially other very closely related species (close enough to align to human). Is this correct?
2. The authors mention using the tool for RNA-Seq (L247) by suggesting the use of abundantly expressed genes. There are potential problems with this approach where some artefacts and biases affecting this type of data would be difficult to correct with a reduced genome approach (eg rRNA contamination). They do later mention that further work would be required for other techniques (L307) but not in the initial section.
3. One point which isn't particularly clear is why it is useful to avoid the whole dataset alignment for the initial QC. My presumption would be that all of these datasets would eventually be aligned against a reference genome anyway, so what is the motivation for delaying this alignment step. Is this to be able to reject libraries without having to align them, or for cases where an alignment free assembly is being done? Setting out the rationale a little more clearly in the introduction would help clarify this.
4. I had some practical difficulty getting the software to run on our cluster (CentOS 7 based). Firstly, in the quickstart instructions there wasn't any mention of the pre-requisites for the software (they are in the paper and are mentioned slightly later in the full instructions), putting these up front would seem to make sense. On our system we didn't have HTSLIB in the main system library directories so the build failed. There were instructions to add these paths at build time, and this helped, but the build then failed because (I think) the cmake files do not include the -std=c++11 option required to enable the 2011

C++ standard on GCC 7.3.0 (the default compiler on CentOS 7). I'll submit a bug report to the github repository with a full log, but it would help to more clearly state the dependencies up front and to make the compile work out of the box with something as standard as gcc 7 (or clearly state if this is not possible).

Level of Interest

Please indicate how interesting you found the manuscript: Choose an item.

Quality of Written English

Please indicate the quality of language in the manuscript: Choose an item.

Declaration of Competing Interests

Please complete a declaration of competing interests, considering the following questions:

- Have you in the past five years received reimbursements, fees, funding, or salary from an organisation that may in any way gain or lose financially from the publication of this manuscript, either now or in the future?
- Do you hold any stocks or shares in an organisation that may in any way gain or lose financially from the publication of this manuscript, either now or in the future?
- Do you hold or are you currently applying for any patents relating to the content of the manuscript?
- Have you received reimbursements, fees, funding, or salary from an organization that holds or has applied for patents relating to the content of the manuscript?
- Do you have any other financial competing interests?
- Do you have any non-financial competing interests in relation to this paper?

If you can answer no to all of the above, write 'I declare that I have no competing interests' below. If your reply is yes to any, please give details below.

I declare that I have no competing interests

I agree to the open peer review policy of the journal. I understand that my name will be included on my report to the authors and, if the manuscript is accepted for publication, my named report including any attachments I upload will be posted on the website along with the authors' responses. I agree for my report to be made available under an Open Access Creative Commons CC-BY license (http://creativecommons.org/licenses/by/4.0/). I understand that any comments which I do not wish to be included in my named report can be included as confidential comments to the editors, which will not be published.

Choose an item.

To further support our reviewers, we have joined with Publons, where you can gain additional credit to further highlight your hard work (see: https://publons.com/journal/530/gigascience). On publication of this paper, your review will be automatically added to Publons, you can then choose whether or not to claim your Publons credit. I understand this statement.

Yes Choose an item.
