# Supplementary material for: FASTQuick: rapid and comprehensive quality assessment of raw sequence reads
Source: Gigascience. 2021 Jan 29;10(2):giab004. doi: 10.1093/gigascience/giab004 (PMC7844880; doi:10.1093/gigascience/giab004)
Supplement: giab004_Reviewer_2_Report_Original_Submission — Tobias Rausch -- 8/25/2020 Reviewed [file giab004_reviewer_2_report_original_submission.pdf]

Reviewer Report

Title: FASTQuick: Rapid and comprehensive quality assessment of raw sequence reads

Version: Original Submission **Date:** 8/25/2020

Reviewer name: Tobias Rausch

Reviewer Comments to Author:

The authors propose FASTQuick, a method for the rapid quality assessment of sequencing data. The method computes standard sequencing QC metrics, it can detect sample contamination and it can infer the ancestry of a sample. The main idea of the method is the construction of a reduced reference genome around known common SNPs and the use of indexing data structures to quickly filter and then align a small subset of reads to this reduced reference genome. Despite a plethora of published NGS QC tools I do see value in the proposed method: The manuscript provides some interesting ideas and optimised algorithms for rapid sequencing QC at the raw read level - with analysis time being an increasingly critical factor in many large-scale clinical genomic studies. The manuscript is well written and the software appears to work. I had no major installation issues and I could also successfully run the software after a couple of smaller hiccups (see comments below). I did notice, however, some remaining issues and inconsistencies in the reported QC values that need to be fixed. Detailed comments are below.

* The abstract would gain in appeal by clearly stating the current major use case of FASTQuick, which is in my opinion human short-read WGS/WES data. In theory, the method could be adapted to non-human species, long reads or other protocols (ATAC-Seq, RNA-Seq, targeted data, ...) but it remains very unclear how much engineering is required for that.

* The ancestry inference lacks some method details. Is this based on a random subset of 1000 Genomes SNPs or is it using a set of ancestry informative markers (AIMs)? I expect established AIMs to be more robust compared to an arbitrary subset of SNPs for samples outside of 1000 Genomes. An evaluation on clinical samples of known ancestry would be more informative than the current comparison to 1000 Genomes in Table S3.

* PCR duplicates are a major problem for poor quality or low-input samples and the duplicate rate often determines whether a sample can be sequenced deeper or not. This is an essential QC metric and a comparison of this experimental feature of FASTQuick to Picard or QPLOT would strengthen the manuscript.

* The introduction briefly touches upon sample swaps. I frankly expected to see a genotyped VCF file for the selected SNPs in the output of FASTQuick that users can then further analyse with tools such as vcftools relatedness2 to identify sample swaps. This would be a useful feature to add, especially if the random SNP set is large enough to distinguish unrelated, parent-child, siblings and identity relationships (i.e., matched tumor-normal genomes in cancer studies).

* Please clarify in the manuscript what type of contamination FASTQuick can identify. I believe it is cross-sample contamination but not tumor-in-normal contamination or microbial sample contamination?

* Minor issues I noticed while running the software are below. For new users it might help to provide the reference files (dbSNP, hs37d5) already in the right format as a resource bundle for download.
(a) Options are called --fastq_1 and --fastq_2 but readme states fastq1 and fastq2
(b) dbSNP reference file needs to be bgzip compressed + tabix-indexed but downloaded file is gzip compressed
(c) hs37d5.fa needs to be bwa-indexed
(d) The output prefix is not used for FinalReport.html
(e) It wasn't clear to me how to run FASTQuick in a UNIX pipe as suggested in the discussion.
* On some test samples, the reported QC results were mostly in-line with our standard QC tools (FastQC, Alfred, VerifyBamID, vcftools relatedness2). Remaining issues:
(a) There is an apparent mismatch between the estimated read mapping rate and the reported number of unmapped reads (NumOfUnmappedReads) in the html produced by the software. For instance on one sample, I got 5% unmapped reads but ~0.99 mapping rate (the latter is the correct estimate).
(b) The accessible genome size was totally off sometimes for WGS human samples: "Total Accessible Genome Size 4607026"
(c) It is unclear to me how to interpret "Estimated Percentage of Accessible Genome Covered -4.00405e+14%"
(d) I often got values > 1 for Depth1, Depth2 ... fractions. Please clarify.
* Minor typos
(a) We chose k=3 based on our experiment based on empirical observations
(b) y=2-2000 should be y=2x-2000 (S1 caption)
* The order of S1 and S3 should be changed because S3 introduces the concept of MaxInsertSize that is used in S1.
* The Figure font size in Figure 1 is too small to be readable.

Level of Interest

Please indicate how interesting you found the manuscript: Choose an item.

Quality of Written English

Please indicate the quality of language in the manuscript: Choose an item.

Declaration of Competing Interests

Please complete a declaration of competing interests, considering the following questions:

- Have you in the past five years received reimbursements, fees, funding, or salary from an organisation that may in any way gain or lose financially from the publication of this manuscript, either now or in the future?

- Do you hold any stocks or shares in an organisation that may in any way gain or lose financially from the publication of this manuscript, either now or in the future?
- Do you hold or are you currently applying for any patents relating to the content of the manuscript?
- Have you received reimbursements, fees, funding, or salary from an organization that holds or has applied for patents relating to the content of the manuscript?
- Do you have any other financial competing interests?
- Do you have any non-financial competing interests in relation to this paper?

If you can answer no to all of the above, write 'I declare that I have no competing interests' below. If your reply is yes to any, please give details below.

I declare that I have no competing interests.

I agree to the open peer review policy of the journal. I understand that my name will be included on my report to the authors and, if the manuscript is accepted for publication, my named report including any attachments I upload will be posted on the website along with the authors' responses. I agree for my report to be made available under an Open Access Creative Commons CC-BY license (http://creativecommons.org/licenses/by/4.0/). I understand that any comments which I do not wish to be included in my named report can be included as confidential comments to the editors, which will not be published.

Choose an item.

To further support our reviewers, we have joined with Publons, where you can gain additional credit to further highlight your hard work (see: https://publons.com/journal/530/gigascience). On publication of this paper, your review will be automatically added to Publons, you can then choose whether or not to claim your Publons credit. I understand this statement.

Yes Choose an item.
